# Heat-shock and methyl-jasmonate: The cultivar-specific responses of pepper plants

**DOI:** 10.3389/fpls.2022.1014230

**Published:** 2022-09-23

**Authors:** Ginés Otálora, María Carmen Piñero, Jacinta Collado-González, Amparo Gálvez, Josefa López-Marín, Francisco M. del Amor

**Affiliations:** Department of Crop Production and Agri-Technology, Murcia Institute of Agri-Food Research and Development (IMIDA), Murcia, Spain

**Keywords:** *Capsicum annuun L.*, mineral concentration, polyamines, sugars, sustainable strategic management, thermotolerance

## Abstract

Frequency, intensity and duration heat-related events have profound implications for future food supply through effects on plant growth and development. This concern needs effective and urgent mitigation tools. However, the effectiveness of potential solutions may decrease according to the specific cultivar response rather consider at specie level. The metyl-jasmonates are essential cellular regulators which are involved in pivotal plant development processes and related to confer protection to heat shock. Thus, our aim was to study the response of three pepper cultivars, Agio (Hungarian type), Basque (Chilli type), and Loreto (Lamuyo type), subjected to heat shock (40°C/72 h) and foliarly-sprayed with methyl-jasmonate (MeJA; 100 µmol), and the effects on several physiological traits. Our results show that despite the important differential impact of heat shock caused on each cultivar, MeJA application did not affect gas exchange, chlorophyll A concentration or efficiency of the photosystem in these cultivars. However, P concentration was reduced when MeJA was applied to Basque chilli, and a significant effect on leaf carbohydrates concentration was observed for Agio and Loreto. Moreover, Agio was the only cultivar in which the amino-acid profile was affected by MeJA under heat shock. Under that condition, putrescine increased for all cultivars, whist the effect of MeJA was only observed for spermine and histamine for Agio and Loreto. Thus, the results indicated that the ameliorative impact of MeJA on this stressor was clearly influenced by cultivar, revealing specific traits. Thus, these results could be used as valuable tools for the characterization of this intraspecific tolerance to heat shock during the vegetative growth stage of pepper.

## Introduction

The current climate crisis demands challenging solutions from agronomists, who have to cope with the new requirements of food and nutrition security, sustainability, and economic welfare (Dhankher and Foyer, 2018). However, the increase in food production under the context of non-optimal climate conditions for crop production (as a result of the increase in frequency and intensity of heat waves), may exceed the physiological thresholds of tolerance of many crops (Russo et al., 2014; Christidis et al., 2015). In Europe’s ecosystem, the gross primary production was reduced by 30% during the heatwave in the summer of 2003 (Ciais et al., 2005), while in the Russian heat wave in summer 2010, this impact was aggravated and estimated at 50% (Bastos et al., 2013). Consequently, new studies to increase product yield whilst maintaining quality are urgently required. Thus, this forces farmers to look for new and more efficient technologies in crop management along with more resilient crop varieties. In fact, changing cultivars could be a useful strategy for climate crisis (Waha et al., 2013), although ignoring cultivar effects, especially for heat stress intensity, could provide biased results and introduce errors in the climate impact assessment results (Rezaei et al., 2018). Presently, it is becoming more frequent to read reports about significant reductions in major crops worldwide (Tito et al., 2018), in which the sensitivity to high temperatures is not only dependent on stress intensity and duration but greatly varies among species (Weng and Lai, 2005) and cultivars (Camejo et al., 2005; Balla et al., 2019). Thus, this stressor can differentially affect plant development and yield and quality, comprising molecular, biochemical, physiological, and morphological alterations (Zandalinas et al., 2018), with their identification and quantification being of paramount importance to achieve the required higher tolerance.

Methyl jasmonate (MeJA) is a natural plant growth regulator that was found to be active in many physiological processes. For example, it can induce some specific proteins that are similar to those induced by heat shock (Weidhase et al., 1987). It was recently indicated that both MeJA biosynthesis and signaling are required for thermotolerance in Arabidopsis (Clarke et al., 2009; Kazan, 2015), thus underlining the importance of the exogenous application of MeJA to confer heat tolerance to horticultural crops. On the other hand, heat stress (HS), defined as short-term temperature increases above the optimum range (Wahid et al., 2007) threatens crop productivity in many areas of the world given the prediction of extreme conditions, and urgently highlights the lack of readiness of our current crop genotypes to cope with future climate extremes (Chavan et al., 2019). Heat shock stress has a complex effect on crops, and to further improve sweet pepper plant tolerance, it is necessary to evaluate and characterize the various mechanisms involved in this crop’s tolerance. However, not enough research has been conducted to compare the main abiotic stress-tolerance traits of sweet pepper cultivars. This crop has been pointed out as sensitive to heat stress, which affects yield and fruit quality (Mateos et al., 2013). Thus, the assessment of heat tolerance in this crop is important in climate-response breeding programs.

Sweet pepper is an important crop species in the Mediterranean-climate areas worldwide, which are some of the climate regions most impacted by the climate crisis (Woetzel et al., 2020). Therefore, the importance of our research is twofold: to gain insights into the physiological processes of the response of pepper to heat stress in relation to the response to MeJA, and to elucidate the way in which these responses are modulated by cultivar. Consequently, we studied photosynthesis and chlorophyll fluorescence, mineral content, carbohydrates, polyamines, and the amino acid profile. The information gained about the role of these traits could establish new ways for managing plant resilience to severe heat waves for this crop.

## Material and methods

### Plant material, growth conditions, and treatments

Three pepper cultivars (*Capsicum annuum* L.) Basque chilli (Chilli pepper type; Ramiro Arnedo Spain S.A.U.), Loreto (Lamuyo type; Syngenta Spain S.A.U.) and Agio (Hungarian type, Nunhems Spain S.A.U.) were obtained from a commercial nursery. Homogeneous pepper seedlings were selected and transplanted into black 5 L containers filled with coconut coir fiber (Pelemix, Alhama de Murcia, Murcia, Spain). Irrigation was supplied by self-compensating drippers (2 L h^-1^). Nutrient solution drainage was monitored to avoid salt accumulation and ionic interference in the rhizosphere, and fresh nutrient solution was provided with minimum drainage of 35%. The experiment was carried out in a climate chamber, as described in del Amor et al. (2010).The acclimation stage lasted 18 days, and the environmental conditions were 60% relative humidity, 16/8 h day/night photoperiod, 26/18°C, and photosynthetically active radiation (PAR) of 250 μmol m^-2^ s^-1^. The nutrient solution (modified Hoagland solution) contained the following minerals: $NO_{3}^{-}:12.0$ ; $H_{2}PO_{4}^{-}:1.0$ ; $SO_{4}^{2-}:3.5$ ; K^+^:7.0; Ca^2+^:4.5; Mg^2+^:2.0. Plants were subjected to heat stress for 72 h in the climate-controlled chamber and the temperature was increased to 40/33/28°C for 12/4/6h and 18/8h day/night photoperiod. At the beginning of the stress period, half of the plants were homogenously sprayed a solution containing MeJA (100µM) and 0.01% Tween-20 as the surfactant. Phenotype picture (control plants) is provided in **Supplementary Figure 1**. Six plants were analyzed per each treatment (sprayed and non-sprayed and heat treatments for 72 h).

### Gas exchange

The net CO_2_ assimilation (ACO_2_), internal [CO_2_] (Ci), transpiration rate (E), and stomatal conductance (gs) were measured in the youngest fully-expanded leaf of each plant, using a CIRAS-2 (PP system, Amesbury, MA) with a PLC6 (U) Automatic Universal Leaf Cuvette, measuring both sides of the leaves. The cuvette provided light (LED) with a photon flux of 1,300 m-2 s-1, 360 or 800 μmol mol-1 CO2, a leaf temperature of 25°C, and 75% relative humidity. The intrinsic water use efficiency (WUE) of leaf gas exchange was calculated from the gas exchange data as ACO_2_/E, where ACO_2_ is the carbon assimilated through photosynthesis, and E is the amount of water lost *via* transpiration.

### Chlorophyll content and fluorescence

Chlorophylls were extracted from samples of the youngest leaf with N,N dimethylformamide, for 72 h, in darkness at 4°C. Subsequently, the absorbance was measured in a spectrophotometer at 750, 664, and 647 nm, and the quantities of Chlorophylls a (Chl a), b (Chl b), and a+b (Chl a+b) were calculated according to the method by Porra et al. (1989). On the leaf used for gas exchange, the dark-adapted maximum fluorescence (Fm) and minimum fluorescence (Fo), and the light adapted, steady-state chlorophyll fluorescence (F) and maximum fluorescence (Fm′) were measured with a portable modulated fluorometer, model OS-30P (Opti-Science, USA). The ratio between the variable fluorescence from a dark-adapted leaf (Fv) and the maximal fluorescence from a dark-adapted, youngest fully-expanded leaf (Fm) – called the maximum potential quantum efficiency of photosystem II (Fv/Fm) - was calculated. A leaf clip holder was placed on each leaf to maintain dark conditions for at least 30 min before reading.

### Mineral composition

The leaf anion concentrations were determined in dry matter, as previously described by Piñero et al. (2016). The anions were analyzed in an ion chromatograph (Metrohm 861 Advanced Compact IC; Metrohm 838 Advanced Sampler); the column used was a Metrohm Metrosep A Supp7 250/4.0 mm. The cation concentrations were analyzed on lyophilized leaves (0.1 g) according to Otálora et al. (2018), by acid digestion, using an ETHOS ONE microwave digestion system (Milestone Inc., Shelton, CT, USA).

### Sugars content

Leaf sugars (glucose, fructose, and sucrose) were determined in leaves by ion chromatography, using an 817 Bioscan (Metrohm, Herisau, Switzerland) system equipped with a pulsed amperometric detector (PAD) and a gold electrode. The column used was a Metrohm Metrosep Carb 1-150 IC column (4.6 x 250 mm), which was heated to 32°C.

### Polyamines

The polyamines content were determined according to Del Rodriguez et al. (2001), with modifications. Pepper leaf samples (5 g), previously stored at -80°C, were mixed with 5 mL of perchloric acid (5%), homogenized for 2 min, and kept for 1 h under refrigeration with periodic stirring, and centrifuged at 5000 x g for 8 min. The supernatant, containing free polyamines, was placed in plastic jars and kept in a freezer at -20°C until use. Free polyamines were benzoylated, 1 mL of sample was taken and mixed with 1 mL of 2 M NaOH and 20 µL of benzoyl chloride. The mixture was stirred in a vortex mixer for 15 s and allowed to rest for 20 min at room temperature. Subsequently, 4 mL of a saturated sodium chloride solution were added, and the system was stirred while 2 mL of diethyl ether were added. The system was left to rest for 30 min at -20°C. Then, 1 mL of the diethyl ether phase was taken and evaporated. The residue was re-suspended in 0.5 mL of acetonitrile/water (56/44 v/v). The polyamines present were analyzed with an ACQUITY UPLC system (Waters, Milford, MA, USA) equipped with a UV detector (230 nm) and a reversed-phase column (ACQUITY UPLC HSS T3 1.8 µm, 2.1 x 100 mm), at 40°C. A mixture of acetonitrile/water (42/58 v/v) was used as the elution solvent, with a flow rate of 0.55 mL/min.

### Free amino acids

Free amino acids were extracted from leaves frozen at -80°C. The pepper leaves were analyzed with the AccQ·Tag-ultra performance liquid chromatography (UPLC) method [Waters, UPLC Amino Acid Analysis Solution. Waters Corporation, Milford, MA (2006)] equipped with a fluorescence detector, as described in Piñero et al. (2017). The amino acids measured were (Arg) arginine; (Ala) alanine; (Asp) aspartic acid; (Ser) serine; (His) histidine; (Ile) isoleucine; (Leu) leucine; (Phe) phenylalanine; (Met) methionine; (Pro) proline; (Gly) glycine; (Thr) threonine; (Lys) lysine; glutamic acid (Glu) and (Val) valine.

### Statistical analysis

The data were tested first for homogeneity of variance and normality of distribution. Significance was determined by analysis of variance (ANOVA), and the significance (p ≤ 0.05) of differences between means was tested with Duncan’s Multiple Range Test using Statgraphics Centurion^®^ XVI software (StatPoint Technologies, Inc). Each treatment had six replicates.

## Results

### Leaf gas exchange and chlorophyll fluorescence parameters

**Figure 1:**
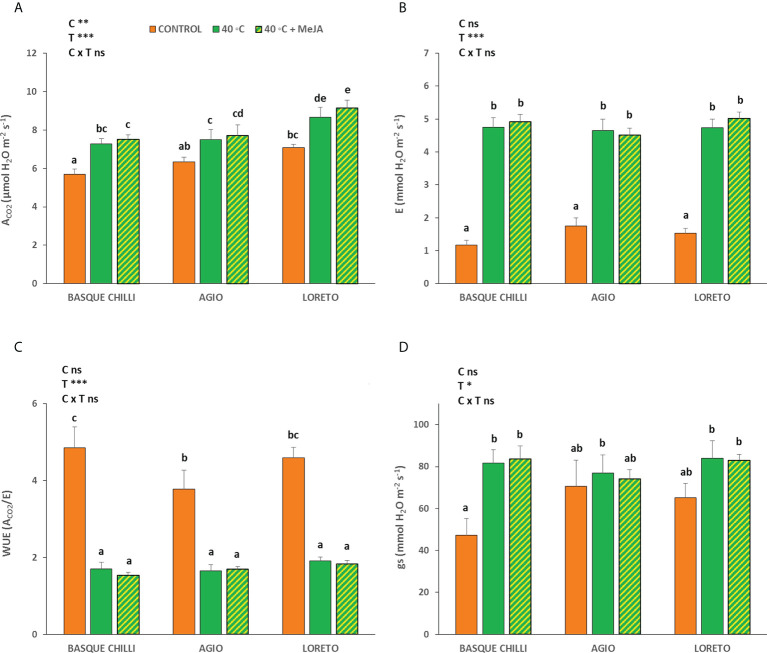
Effects of heat shock and MeJA on **(A)** the net CO_2_ assimilation rate (A_CO2_), **(B)** transpiration (E), **(C)** stomatal conductance (gs), and **(D)** intrinsic water use efficiency (WUE) in leaves of the three pepper cultivars. Values are means ± SE. Different letters indicate significant differences between treatments according to Duncan’s test at the 95% confidence level. The * refers to significant differences at the level of p ≤ 0.05; **p ≤ 0.005; ***p ≤ 0.001; n.s., not significant. C is cultivar; T is temperature.

**Figure 2:**
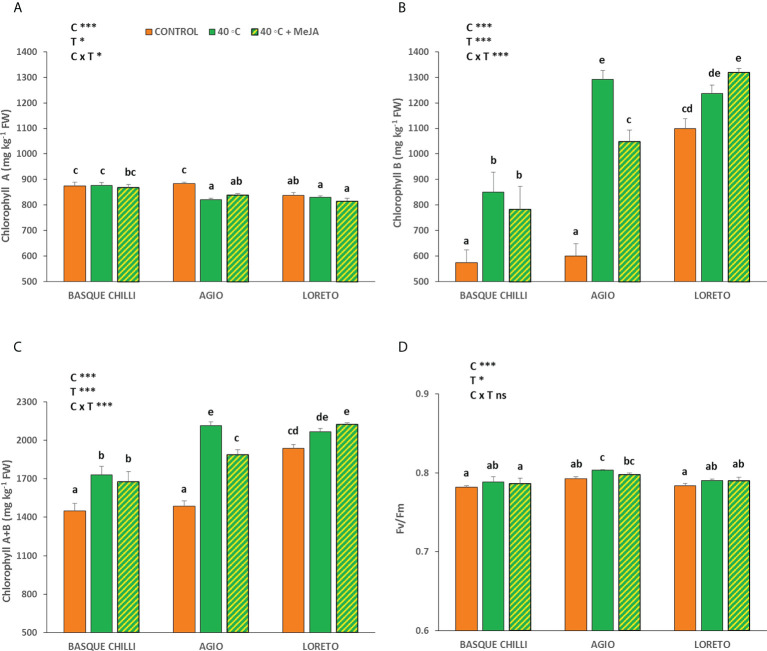
Effects of heat shock and MeJA on **(A)** Chlorophyll a, **(B)** Chlorophyll b, **(C)** total Chlorophylls (a+b), and **(D)** the maximum potential quantum efficiency of photosystem II (Fv/Fm) in leaves of the three pepper cultivars. Values are means ± SE. Different letters indicate significant differences between treatments according to Duncan’s test at the 95% confidence level. The * refers to significant differences at the level of p ≤ 0.05; ***p ≤ 0.001; n.s., not significant. C is cultivar; T is temperature.

The effect of the imposed heat stress was different depending of the cultivar studied. The induced heat shock wave produced a significant increase in the net photosynthetic rate for all cultivars, with this effect being cultivar-dependent (**Figure 1A**). Thus, the impact of the stress was significantly higher for the Basque chilly, as shown by the increased leaf photosynthesis of 27.7%, as compared with the less affected cultivars Hungarian type (Agio) or Lamuyo type (Loreto), with an increase of 22.3% and 18.3%, respectively. The application of MeJA before stress had a relatively minor effect on the three cultivars, but Loreto showed the highest photosynthetic values. The response for leaf stomatal conductance and transpiration, and consequently intrinsic water use efficiency, were less affected among cultivars, as compared to the data observed for leaf photosynthesis, with the effect of the cultivar being not significant. As expected, the transpiration rate (**Figure 1B**) was higher when heat stress was applied, although the effects of the cultivar and MeJA were not significant. Accordingly, intrinsic water use efficiency (**Figure 1D**) had the same effect. The response for leaf stomatal conductance (**Figure 1C**) with increasing temperature was not affected in the three cultivars. The impact of heat stress on chlorophyll A (Chll A) concentration (**Figure 2A**) was remarkable in Agio, but not for Basque chilli or Loreto. However, chlorophyll B (Chll B) (**Figure 2B**) was clearly affected in the three cultivars to a large extent. Thus, when cultivars were subjected to heat stress for 72 h, a significant leap was observed for Chll B in Basque chilli and Agio (47.9% and 115.1%) although this was lower for Loreto (12.4%). The application of MeJA caused no impact on Basque chilli or Loreto, although Agio showed the highest response (reducing the observed increase in Chll B due to heat stress to 74.5% with respect the non-stressed plants). In addition, a similar effect as Chll B was found for total chlorophylls (**Figure 2C**), as Chll B, was a major component of this parameter. Moreover, we found a weak effect of both treatments on chlorophyll fluorescence (**Figure 2D**) and only Agio showed a slight increase (1.3%) with heat shock, although the application MeJA did not have a significant effect.

### Leaf mineral concentration

Leaf nitrate concentration significantly increased in the three studied cultivars after heat stress, with Basque chilli being the most responsive, with an increase of 26.7%, followed by Loreto (19.4%) and Agio (18.4%) with respect of non-stressed plants for the same cultivar (**Table 1**). The concentrations of phosphates, sulfates, and chlorides concentration were more affected according to cultivar. Loreto showed a clearly distinctive pattern with heat stress, with a significant reduction observed for phosphates and chlorides, whilst the increase in sulfates was more attenuated as compared with Basque chilli and Agio. Loreto was also the cultivar where the addition of MeJA resulted in a reduction of nitrate concentration as compared with stressed plants, without significant differences observed with respect to non-stressed plants. The concentration of cations varied among cultivars (**Tables 2**, **3**). Control plants of Basque chilli showed the lowest Ca concentration, whilst Loreto had the highest P and Mg concentrations. When subjected to heat stress, the Basque chilli cultivar was less affected with respect to the cation concentrations, but interestingly, MeJA attenuated the accumulation of P after heat stress. Agio had a similar response for P, but this effect was not observed for Loreto, which prompted the highest Na concentration in the leaves. Micronutrients (**Table 3**) concentrations were markedly affected, where B concentration increased by 17.6% and 33.8%, respectively, in Basque chilli and Agio, by the heat stress. Loreto showed differences for Cu and Zn (which increased 50.8% and 15.84%), while Mn concentration was affected in Agio and Loreto (42.3% and 82.7%), although Basque Chilli did not show a significant difference for this macronutrient after heat stress. MeJA did not affect the response of leaf micronutrients in the pepper plants subjected to heat stress.

**Table 1:** Effects of heat shock and MeJA on leaf anion concentration (g kg^-1^ DW) of the three pepper cultivars.

Cultivar	Treatment	Chlorides	Nitrates	Phosphates	Sulfates
BASQUE CHILLI	Control	1.06 ± 0.10 c	39.29 ± 1.12 abc	14.05 ± 0.40 bc	6.08 ± 0.19 a
	40°C	0.96 ± 0.06 bc	49.77 ± 1.88 de	17.72 ± 0.56 d	7.17 ± 0.36 abc
	40°C + MeJA	0.97 ± 0.03 bc	49.06 ± 2.09 de	13.66 ± 0.88 bc	6.86 ± 0.58 ab
AGIO	Control	1.01 ± 0.08 bc	43.90 ± 4.02 cd	13.95 ± 2.10 bc	6.82 ± 0.78 ab
	40°C	1.17 ± 0.12 c	51.98 ± 1.34 e	16.94 ± 0.91 cd	8.44 ± 0.24 c
	40°C + MeJA	0.99 ± 0.07 bc	53.65 ± 1.90 e	16.40 ± 0.70 cd	8.50 ± 0.30 c
LORETO	Control	0.76 ± 0.07 b	34.46 ± 1.43 a	16.54 ± 2.10 cd	7.12 ± 0.20 abc
	40°C	0.48 ± 0.05 a	41.15 ± 1.37 bc	11.18 ± 0.79 ab	7.60 ± 0.41 bc
	40°C + MeJA	0.44 ± 0.05 a	36.74 ± 1.82 ab	9.71 ± 1.02 a	6.67 ± 0.52 ab
ANOVA	Cultivar (C)	***	***	**	*
	Temperature (T)	ns	***	ns	*
	C x T	ns	ns	***	ns

Values are means ± SE. Different letters indicate significant differences between treatments according to Duncan’s test at the 95% confidence level. The * refers to significant differences at the level of p ≤ 0.05; **p ≤ 0.005; ***p ≤ 0.001; n.s., not significant.

**Table 2:** Effects of heat shock and MeJA on leaf cation concentration (g kg^-1^ DW) of the three pepper cultivars.

Cultivar	Treatment	Ca	K	Mg	Na	P
BASQUE CHILLI	Control	9.28 ± 0.22 a	57.48 ± 1.40 a	5.08 ± 0.14 a	0.15 ± 0.01 a	4.72 ± 0.13 a
	40°C	10.28 ± 0.27 a	61.02 ± 1.87 ab	5.71 ± 0.29 abc	0.16 ± 0.01 a	5.92 ± 0.08 cd
	40°C + MeJA	9.90 ± 0.28 a	59.73 ± 1.25 ab	5.54 ± 0.31 ab	0.18 ± 0.01 ab	5.05 ± 0.29 ab
AGIO	Control	14.36 ± 0.68 cd	58.86 ± 2.49 ab	5.04 ± 0.28 a	0.19 ± 0.02 abc	5.20 ± 0.40 ab
	40°C	15.33 ± 0.69 de	59.59 ± 1.58 ab	5.60 ± 0.25 abc	0.20 ± 0.01 abc	5.89 ± 0.17 cd
	40°C + MeJA	16.42 ± 0.78 e	62.88 ± 2.15 ab	5.67 ± 0.24 abc	0.23 ± 0.02 bcd	5.44 ± 0.12 bc
LORETO	Control	13.90 ± 0.71 bcd	62.41 ± 1.26 ab	6.05 ± 0.21 bc	0.24 ± 0.01 cde	5.92 ± 0.32 cd
	40°C	13.34 ± 0.58 bc	64.01 ± 0.96 b	6.41 ± 0.17 c	0.27 ± 0.01 de	6.88 ± 0.10 e
	40°C + MeJA	12.40 ± 0.34 b	63.73 ± 1.49 b	6.05 ± 0.17 bc	0.30 ± 0.02 e	6.49 ± 0.17 de
	Cultivar (C)	***	*	**	***	***
ANOVA	Temperature (T)	ns	ns	ns	*	***
	C x T	ns	ns	ns	ns	ns

Values are means ± SE. Different letters indicate significant differences between treatments according to Duncan’s test at the 95% confidence level. The * refers to significant differences at the level of p ≤ 0.05; **p ≤ 0.005; ***p ≤ 0.001; n.s., not significant.

**Table 3:** Effects of heat shock and MeJA on leaf cation concentration (mg kg^-1^ DW) of the three pepper cultivars.

Cultivar	Treatment	B	Cu	Fe	Mn	Zn
BASQUE CHILLI	Control	33.83 ± 1.26 a	7.67 ± 0.72 a	122.12 ± 6.51 ab	12.58 ± 1.13 a	48.83 ± 2.07 a
	40°C	39.77 ± 1.24 b	10.94 ± 0.97 bc	116.05 ± 3.30 a	15.88 ± 1.57 ab	62.75 ± 1.98 b
	40°C + MeJA	40.93 ± 0.58 b	9.95 ± 1.43 abc	114.22 ± 4.98 a	12.02 ± 1.54 a	56.75 ± 4.43 ab
AGIO	Control	31.06 ± 1.54 a	7.18 ± 0.51 a	118.36 ± 4.36 ab	12.54 ± 1.90 a	66.05 ± 6.17 bc
	40°C	41.55 ± 1.35 b	8.77 ± 0.77 ab	115.89 ± 3.39 a	17.85 ± 1.96 b	76.12 ± 3.52 c
	40°C + MeJA	42.13 ± 0.94 b	8.45 ± 0.67 ab	115.73 ± 2.25 a	18.48 ± 1.33 b	74.58 ± 2.02 c
LORETO	Control	30.43 ± 2.44 a	7.76 ± 0.86 a	125.57 ± 8.76 ab	11.31 ± 1.44 a	77.38 ± 3.98 c
	40°C	33.56 ± 0.64 a	11.70 ± 0.63 c	133.09 ± 4.09 b	20.66 ± 0.76 b	99.06 ± 3.58 d
	40°C + MeJA	32.50 ± 0.70 a	12.40 ± 0.76 c	128.41 ± 6.89 ab	19.05 ± 2.22 b	89.64 ± 3.92 d
	Cultivar (C)	***	*	*	*	***
ANOVA	Temperature (T)	***	***	ns	***	***
	C x T	*	ns	ns	ns	ns

Values are means ± SE. Different letters indicate significant differences between treatments according to Duncan’s test at the 95% confidence level. The * refers to significant differences at the level of p ≤ 0.05; ***p ≤ 0.001; n.s., not significant.

### Leaf sugars concentration

**Figure 3:**
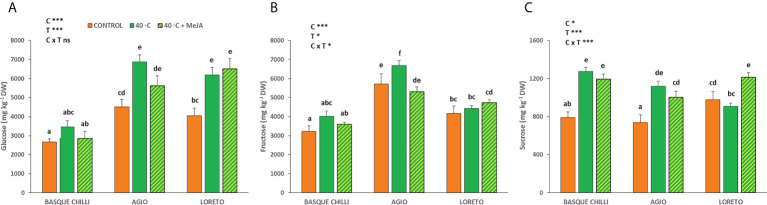
Effects of heat shock and MeJA on leaf carbohydrate concentration [**(A)** glucose, **(B)** fructose and **(C)** sucrose] of the three pepper cultivars. Values are means ± SE. Different letters indicate significant differences between treatments according to Duncan’s test at the 95% confidence level. The * refers to significant differences at the level of p ≤ 0.05; ***p ≤ 0.001; n.s., not significant. C is cultivar; T is temperature.

The heat stress differentially impacted the cultivars, and the leaf carbohydrates concentrations varied by cultivar (**Figure 3**). Glucose concentration was not altered by heat in Basque chilli, but Agio and Loreto increased its concentration in a similar way (52.7% and 52.5%) as compared with the non-stressed plant of the same cultivar. However, fructose was only increased in Agio (16.8%) when heat shock was applied. On the other hand, sucrose was noticeably impacted in Basque chilli, which was the only sugar affected in this cultivar after stress (61% increase). The behavior of sucrose for Agio was similar to Basque chilli, while Loreto experienced a significant increase for this carbohydrate when MeJA was applied.

### Polyamines

**Figure 4:**
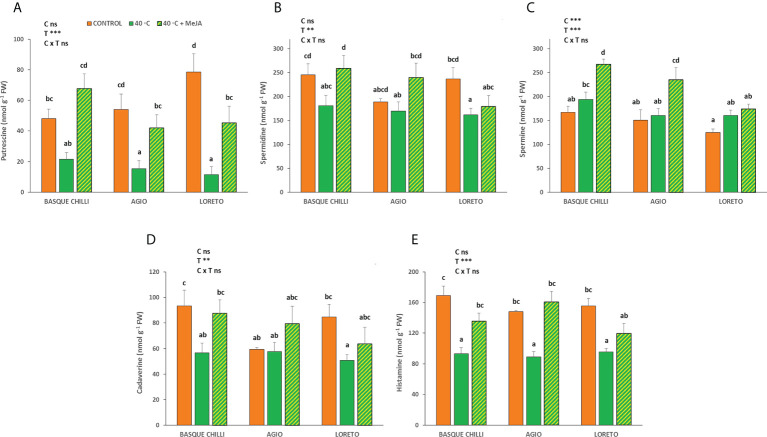
Effects of heat shock and MeJA on leaf polyamines (**(A)** putrescine, **(B)** spermidine, **(C)** spermine, **(D)** cadaverine and **(E)** histamine) of the three pepper cultivars. Values are means ± SE. Different letters indicate significant differences between treatments according to Duncan’s test at the 95% confidence level. The ** refers to significant differences at the level of p ≤ 0.005; ***p ≤ 0.001; n.s., not significant. C is cultivar; T is temperature.

MeJA had an important effect on the putrescine concentration and showed similar results for all cultivars (**Figure 4**). Thus, while heat stress significantly reduced putrescine by 55.1%, 71.5%, and 85.5% for Chilli, Agio and Loreto, with respect non-stressed plants, the application of MeJA to the stressed ones remarkably increased putrescine concentration by 213.9%, 174.3% and 297.6%, respectively. The results for spermidine were more attenuated and only the Basque chilli showed a significant effect on the MeJA application. Spermine concentration also increased after MeJA application as compared with stressed plants without MeJA, but only for Basque chilli and Agio (37.9% and 46.7%), with no significant differences observed for Loreto. Cadaverine showed a pattern similar to spermidine. Histamine concentration was also reduced after 72 h of the imposed stress by 44.8%, 39.2%, and 38.5% for Basque chilli, Agio and Loreto, respectively, but the application of MeJA before stress restored them to non-stressed values.

### Free amino acids

**Figure 5:**
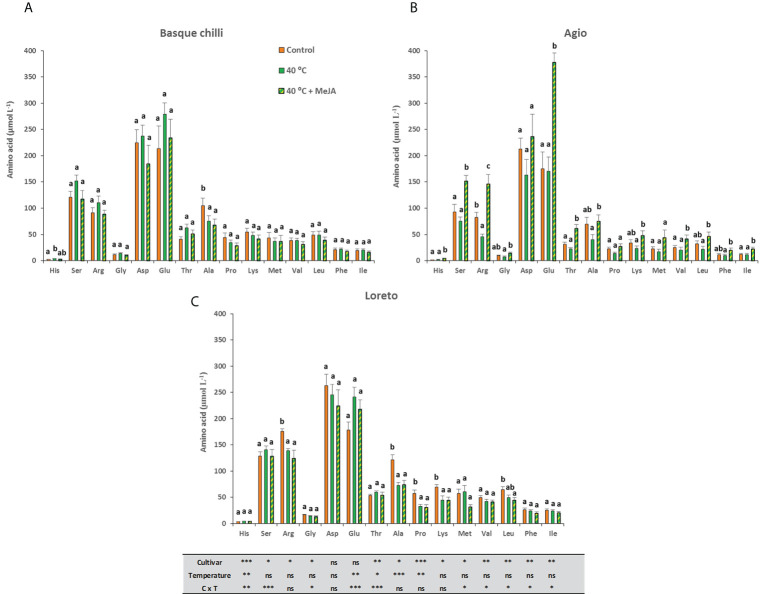
Effects of heat shock and MeJA on leaf amino acids concentration (µmol L^-1^) of the three pepper cultivars [**(A)** Basque chilli, **(B)** Agio and **(C)** Loreto]. Values are means ± SE. Different letters indicate significant differences between treatments according to Duncan’s test at the 95% confidence level. The * refers to significant differences at the level of p ≤ 0.05; **p ≤ 0.005; ***p ≤ 0.001; n.s., not significant.

The leaf amino acid concentration in the studied cultivars had a broad and diverse response to heat stress. Thus, the Basque chilly response was the most attenuated to both heat stress and MeJA (**Figure 5A**), whilst the Agio (**Figure 5B**) and Loreto (**Figure 5C**) cultivars showed a more specific response. Agio showed a remarkable effect after the application of MeJA before heat stress and the amino acids histidine, serine, arginine,threonine, valine, and isoleucine were significantly affected and as compared with stressed plants, the concentrations in the leaf increased by 144.5%, 102.5%, 220.3%, 174.5%,104.6%, and 105.5%, respectively. These increases were also very significant although lower when these were compared with the MeJA amendment and with the non-stressed plants. In the case of Loreto, the application of MeJA had a very reduced effect, with the response mainly attributed to the heat stress alone. In this case, we observed differences for Arginine, Alanine, Proline, and Lysine, and the decreases (instead of increases reported for Agio) were (compared with the non-stressed ones) 20.9%, 40.1%, 46.4%,35.0%.The most abundant amino acids found in the three untreated pepper cultivars were Ser,Arg, Asp, Ala and Glu, representing around 70%, 75%, and 73% of the total amino acids in Basque chilli, Agio, and Loreto, respectively. MeJA applied to Basque chilli and Loreto pepper plants had a lesser effect on the content of these compounds than in Agio pepper plants.

**Table 4** shows the percentage of variance attributable to temperature, variety, and their interaction on pepper amino acid content. Temperature × variety was the most dominant factor for the concentrations of Ser, Arg, Gly, Asp, Thr, Met, Ile, and total amino acids.Temperature was the most dominant factor of variation for the concentrations of Ala and Glu. Variety was the most dominant factor of variation for the concentrations of His, Pro, Lys, Val, Leu, and Phe.

**Table 4:** Percentage of variance attributable to temperature, variety and their interaction for each amino acid of the three pepper cultivars.

	Temperature (%)	Variety (%)	Temperature x variety (%)	Residual (%)
His	17.26 **	26.54 ***	19.47 **	36.71
Ser	4.80 NS	12.13 *	36.14 ***	46.92
Arg	5.33 NS	35.80 ***	38.98 ***	19.87
Gly	0.87 NS	20.03 *	24.04 *	55.05
Asp	1.65 NS	7.08 NS	12.68 NS	78.57
Glu	19.83 **	3.25 NS	37.97 ***	38.93
Thr	10.12 *	18.83 **	30.73 ***	40.29
Ala	25.05 ***	16.56 *	12.07 NS	46.31
Pro	17.57 **	29.70 ***	13.01 NS	39.70
Lys	9.66 NS	17.55 *	16.87 NS	55.90
Met	0.67 NS	20.80 *	26.88 *	51.64
Val	2.32 NS	23.34 **	20.48 *	53.84
Leu	4.17 NS	23.68 **	20.22 *	51.92
Phe	0.33 NS	26.36 **	17.89 *	55.41
Ile	0.47 NS	22.13 **	23.07 *	54.32
Total aas	11.20 *	13.56 **	42.68 ***	32.55

Statistically significant at *p ≤ 0.05, **p ≤ 0.01 and ***p ≤ 0.001, respectively. NS, not significant.

## Discussion

Our data showed that the imposed heat stress had a positive effect on increasing leaf net CO2 assimilation rate for all the studied cultivars. Griffin et al. (2004) reported that Rubisco, the central enzyme of photosynthesis, is disrupted if the temperature increases from 35°C, stopping the photosynthetic process. That reduction in the photosynthetic process has been attributed to the inhibition of photosystem II in the thylakoid membrane, which impairs chlorophyll fluorescence (Chen et al., 2017) while decreasing stomatal conductance and transpiration (Fahad et al., 2016). However, these effects attributable to stomatal closure and/or inhibition of the inactivation of Rubisco, and the loss of chlorophyll contents (Ristic et al., 2008) were not detected in our experiment with pepper at a higher temperature, which increased or maintained Chlorophylls (a+b) content whilst increasing leaf transpiration, although WUE was reduced. The resilience of the photosynthetic machinery to high temperatures was also reported in our previous work with sweet pepper and elevated CO2 concentration during the vegetative growth stage (Pérez-Jiménez et al., 2019). At this point, we agreed with Rizhsky et al. (Rizhsky et al., 2002) who also indicated that high temperatures do not impair photosynthesis along with the intensification of the stomatal conductance. However, Pagamas and Nawata (2008) and Lee et al. (2018) pointed out the beneficial effect of high temperature on vegetative parameters. Interestingly we observed a differential response for Agio, where chlorophyll B clearly increased in concentration by heat (a similar response for all cultivars), but chlorophyll A was reduced. In squash, Ara et al. (2013) reported that Chl. B degraded more drastically under high temperature than Chll A, with the destruction of photosynthetic pigments under heat stress providing a clue for determining thermotolerance. However, Zhou et al. (2015), when screening heat tolerance for tomato genotypes, observed that heat stress led to a significant increase in the content of Chll A, Chl. B, and total chlorophyll contents in the heat-tolerant group, while in the heat-sensitive group, a significant decrease in the pigment contents was observed. In our experiment only Agio was affected (reduced Chll A content), and despite Chll A being a primary electron donor in the electron transport chain and the primary pigment in photosynthesis, photosynthesis was not affected after heat stress in this cultivar. Moreover, an increase in Chll B was identified for all cultivars, probably attributed to an adaptation to high temperature for this species. The application of MeJA did not increase any gas exchange parameters beyond the temperature effects alone.The concentrations of leaf mineral nutrients were different under non-stressed conditions for the three cultivars. This is in agreement with the results from Ropokis et al. (2018), which uptake of nutrients was different under the same water regime, nutritional supply, and environmental conditions. Under heat stress, leaf NO3-concentration was significantly increased, although this increase was different depending on the cultivar, with Loreto being the least responsive. However, MeJA did not alter the response of the stressed cultivars. Huang et al. (Huang et al., 2017) indicated that exogenously applied jasmonate was related with inhibition of primary root growth. The effect of MeJA on root morphology could trigger alterations in nutrient uptake, especially under this stress condition, although a marginal effect was found for these pepper cultivars. This smaller effect can be attributable to the reduced time (72h) of the application to harvest, which reduced the effect on the accumulated growth. Sugar can act as signal molecules in the leaves, playing a pivotal role in regulating development, and triggering leaf senescence (Van Doorn, 2008; Reinbothe et al., 2009). Sugars can also impair photosynthesis when overcoming threshold concentrations (Zhang and Zhou, 2013). In tomato, the maintenance or higher levels of non-structural carbohydrates in mature leaves were associated with heat tolerance under short-term heat stress (Zhou et al., 2017). Sucrose, the final product of photosynthesis, can either act as a signaling molecule or become a reactive oxygen species scavenger, at low or high concentrations, respectively (Frank et al., 2009), with the latter protecting the plants from cellular damages and degradation of pigments such as chlorophylls. Our data showed that heat shock had an impact on the plants, especially for the Agio cultivar, in which glucose, fructose and sucrose were clearly reduced when MeJA was applied; however, an increase in sucrose concentration was observed in Loreto. Such alterations in carbohydrate concentration did not impair CO2-fixation. Gómez et al. (2010) pointed out in tomato that MeJA produced a rapid increase in leaf export of photosynthates, which did not occur in pepper.

Recent works have indicated that endogenous polyamines could participate in sustaining membrane integrity in cauliflower, as protectors against damage under stress conditions, through the stabilization of cell membranes (Collado-González et al., 2021a; Collado-González et al., 2021b), and therefore, they could have an effect in delaying leaf-related-senescence disorders. As previously indicated for other species, the collateral effect of MeJA associated with early senescence, can be effectively counteracted by the increase in the concentration of endogenous polyamines in specific pepper cultivars. Thus, in our stress conditions, this remarkable effect of MeJA can partly mitigate further leaf damage. A decrease in amino acid contents has also been observed for tomato (Gómez et al., 2010) and Brassica rapa leaves (Liang et al., 2006) after treatment with the foliar application of MeJA, with no stress conditions. Our data showed, after thermal stress, that MeJA application only affected the Agio cultivar, which clearly increased the concentration of their leaf amino acids. Consequently, a cultivar-dependent response was observed for pepper after the application of MeJA. (Garde-Cerdán et al., 2016) indicated that the amino acid profile of grapes was altered after the application of MeJA. In this study, we found a smaller decrease in the total amino acid content under heat stress in Agio and Loreto as compared to Basque chilli. Also, the effect of MeJA increased total amino acid content in the Agio variety alone. The differential accumulation of free amino acids could be associated with the genetic variations of heat tolerance in pepper plants. Thus, the effects of MeJA on pepper amino acid concentration were conditioned by variety. Gutiérrez-Gamboa et al. (2018) showed that grape variety had a higher effect on must amino acid composition than the elicitation through MeJA and yeast extract treatments and their interaction (variety x treatment).

## Conclusions

Horticultural production is one of the most vulnerable agrosystems to the temperature impacts of climate change, and the effectiveness of mitigation strategies (such as the application of elicitors) against to this constraint needs to be widely contrasted. Our work shows the differential effects of pepper cultivars to heat stress with the application of MeJA, and also describes and examines the physiological traits involved in heat tolerance and the physiological mechanics affected. However, more studies are needed to screen a wider range of stress intensities and durations during the vegetative phase of growth and throughout the other developmental stages. To date, very few studies have addressed the specific differences of cultivars to high temperature and MeJA to show their specific responses. To this end, our study sought to investigate the physiological traits involved in that specific response to provide new knowledge for the development of new cultivars, selected to withstand the impact of heat waves without compromising yields.

## Data availability statement

The raw data supporting the conclusions of this article will be made available by the authors, without undue reservation.

## Author contributions

GO: conceptualisation, methodology, formal analysis, investigation, writing, editing. JG and MP: methodology, and formal analysis. JL-M: supervision. FA: conceptualization, funding acquisition, resources, project administration, supervision. All authors contributed to the article and approved the submitted version. All authors contributed to the article and approved the submitted version.

## Funding

This work was financed by the European Regional Development Fund (ERDF) 80% – Region de Murcia (FEDER 1420-30).

## Acknowledgments

We thank José Manuel Gambín, Miguel Marín, José Sáez Sironi, and Raquel Roca, for their technical assistance.

## Conflict of interest

The authors declare that the research was conducted in the absence of any commercial or financial relationships that could be construed as a potential conflict of interest

## Publisher’s note

All claims expressed in this article are solely those of the authors and do not necessarily represent those of their affiliated organizations, or those of the publisher, the editors and the reviewers. Any product that may be evaluated in this article, or claim that may be made by its manufacturer, is not guaranteed or endorsed by the publisher.
