# Supplementary figures and images for: Heat-shock and methyl-jasmonate: The cultivar-specific responses of pepper plants
Source: Front Plant Sci. 2022 Sep 23;13:1014230. doi: 10.3389/fpls.2022.1014230 (PMC9539432; doi:10.3389/fpls.2022.1014230)

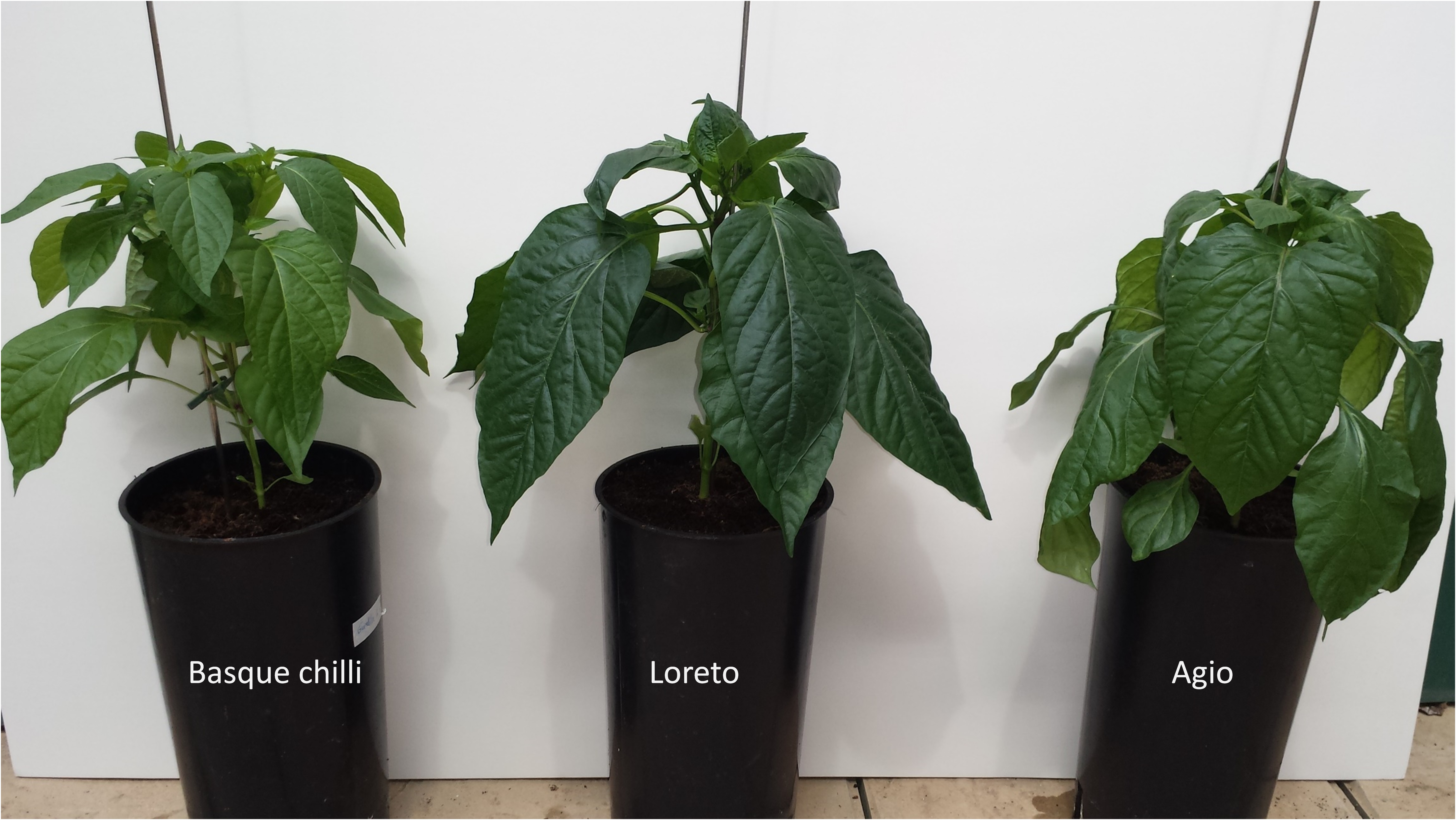

Supplement: Supplementary file 1 [file Image_1.jpeg]
